# Epidemic Cholera and Dysentery at Cincinnati, 1834

**Published:** 1834

**Authors:** 

**Affiliations:** Cincinnati


### The Western Journal

OF THE

### MEDICAL AND PHYSICAL SCIENCES.

JULY, AUGUST, AND SEPTEMBER, 1834.

### ESSAYS AND CASES.

### Art. I.—Epidemic Cholera and Dysentery.

*An Account of the Third Visitation of Epidemic Cholera, in connexion with Dysentery, at Cincinnati, in the summer of* 1834. By the Editor.

#### Historical and Ætiological Remarks.

Called upon, as medical historians, to say something more of the Epidemic of which we have already said so much, we begin by observing, that this third visitation at Cincinnati, was milder than the second, which was less violent and mortal than the first. Thus each succeeding attack has indicated either a diminishing energy in the remote cause, or decreasing aptitude to its action, in the systems of the exposed—probably the latter—possibly both; where, however, the disease had not prevailed before to much extent, it was this year extremely fatal, (as in certain parts of the country,) from which it may be inferred that the virulence of the remote cause, was in fact unabated.

Although our object is the disease in Cincinnati and its vicinity, where it fell under our own observation, it is right that we should notice its chronology and geography on a more extended scale.

It is curious, that while, in 1832, the malady was developed progressively, from the shores of the St. Lawrence to those of the lower Mississippi, appearing on the former early in July—on the latter, near the end of October; its development the next and the present year, was in the reversed order. Thus it was prevalent in the spring in New Orleans, and gradually invaded other towns up to the neighborhood of Cincinnati, where it commenced in July, after it had ceased below; and subsequently broke out at Wheeling, Pittsburgh, and other places higher up in the valley of the Ohio—appearing at a still later period, in the basins of the St. Lawrence and the Hudson.

It is, also, worthy of remark, that all its visitations have been more especially directed upon that zone in North America, which extends from the Gulf of the St. Lawrence to the Gulf of Mexico—from Quebec to New Orleans—in the valleys of the Lakes and the Mississippi; almost every part of which (including both town and country) has, in the course of three years, been invaded. The region of which we speak, comprehends Canada, the western parts of the states of New York, Pennsylvania, and Virginia, the Michigan territory, and the states of Ohio, Indiana, Illinois, Missouri, Kentucky, Tennessee, Mississippi, and Louisiana. It has spread beyond this interior region, in one quarter only. In 1832, it extended into the basins of the Hudson, Delaware, and Susquehana, and partially into that of the Potomac; affecting, more or less, the states of New York, New Jersey, Pennsylvania, Maryland, and Virginia; limited, however, in a great degree, to their maritime cities, and making but little progress in the last mentioned state. It is still more remarkable, that in the third visitation, it should have nearly confined itself to those states—even to a limited portion of two of them, New York and New Jersey. Thus, two great geographical and political sections of the Union, the north-eastern and the south-eastern, have as yet almost escaped. The former comprehends Connecticut, Rhode Island, Massa. chusetts, Vermont, New Hampshire, and Maine. The latter embraces southern Virginia, North and South Carolina, Georgia, Alabama, and Florida. Scattering cases, sometimes considerably numerous, have, it is true, shown themselves, but no where have they been sufficient to constitute the disease an epidemic. As the two great divisions, the north and the south of the maritime states, differ widely in latitude, and manners, customs, pursuits, and modes of living, we may infer that these circumstances do not influence the production of the disease. But is there nothing in their geology, that may have exerted an effect on the spread of the malady? A straight line from Quebec to New Orleans, would almost constitute the south-eastern boundary of the Epidemic, except in one part, where two parallel lines drawn from the valley of the St. Lawrence and Lakes to the sea shore, would reach it at the mouths of the Hudson and James River. Now the region north-west of the first mentioned line is secondary, with extensive diluvial and alluvial deposites, and limestone is the predominant rock. The region northeast of the line running from the St. Lawrence to the city of New York, is almost entirely primitive, and has but small tracts of diluvium; and the region south-west of the line from the Lakes to the mouth of James River, is primary and transition, but has extensive marine alluvions.

These views would seem, at first sight, to indicate a geological influence; but the hypothesis is overturned by the fact, that the cities of New York, Philadelphia, Baltimore, Washington, Richmond, and Norfolk, are all built on primitive rocks (granite and gniess) or on beds of sand and gravel, consisting of the *debris* of primitive formations. It would seem, therefore, that the distribution of the disease, over the United States and Canada, has not depended on the geological structure of their different parts; though as it has visited the cities just enumerated, less frequently and severely than the interior, it may be said to have been more violent over the secondary formations. Whether this connexion was that of cause and effect, we have not as yet the facts necessary to a logical decision. It is proper to wait and see whether the parts heretofore comparatively exempt, may not hereafter be scourged.

It is certainly humbling to the pride of medical science, that its votaries, scattered over three quarters of the globe, and continuing their observations for sixteen years, should have been unable to discover the cause of this wandering pestilence. The theory of cometary influence will not do—that of miasmatic exhalation will not do—that of atmospheric animalcules, no one believes—that of insensible meteoration no body comprehends—and that of contagion is met by the established and undisputed fact, that the disease has repeatedly attacked persons in remote and insulated places, when they had not been subjected to the least communication with the sick, or any thing belonging to them. We are aware that cases of contagious origin, are perpetually cited by the people and a few professional advocates for that theory; but, as we have said, on a former occasion, the malady may, and does occur independently of contagion; it is demonstrated, therefore, that there *is* a cause that is *not* contagion, and although we do not know what it is, we are as little at liberty, according to the rules of philosophical logic, to admit another, as if we did. Moreover, if contagion were the cause, why does not the disease spread into New England and the Carolinas, and why did it appear in the present summer, nearly at the same time, on the banks of the St. Lawrence and Hudson, and the southern shores of Lake Erie, from Cleveland to Detroit? Still further, why has it sorely afflicted certain neighborhoods in Ohio, and left others adjoining untouched? We are, of course aware, that the question may, with equal propriety, be asked, why any other cause, of a general nature, should be active in one village, and produce no effect in another? to which we give this answer, *that we cannot tell*; but add, that we are not called upon to believe in contagion, merely because we have nothing else to rest upon. In denying contagion as the agent by which the spread of Cholera is effected, we are not bound to substitute any other cause, but must with becoming humility confess our ignorance.

In this state of uncertainty, every fact is of importance, and we shall, therefore, proceed to furnish two, which, although not unobserved before, deserve to be recorded again. First, in its reproduction, during the present summer, the Epidemic has arisen, and declined, with the intense heat, commencing in May, and abating, or ceasing entirely, in August; second, in many parts of the country around Cincinnati, it has prevailed much more on the uplands than in the valleys. Even in Cincinnati, it has been more frequent and fatal on the elevated than in the lower parts of the city. Above and below the town, it has prevailed, but in very different degrees. Below, where there is greater depression, and more extensive alluvial deposites, than above, it has been of single prevalence; up the river, in the town of Fulton, it was general and extremely fatal. This village consists of a single street, three miles in length, running between the river and the adjoining hill, at a considerable elevation above high water. The face of the hill is a lime stone cliff, and the houses are built amidst the fragments of rock and marl, that have fallen from it, and have formed a slope to the margin of the stream. There is consequently, no alluvial deposites, no special accumulations of filth, and no stagnant water; still the malady raged violently in this open village, for some time before it appeared in the city, and (although it was prevalent there in the two preceding years) carried off nearly four per cent. of the inhabitants. Was there then no physical circumstance in which Fulton varied from the surrounding places? The only one in which it differed, to its apparent disadvantage, was in its lying in a curve of the hill, with mural precipices to the north, reflecting and reverberating upon it, the rays of the sun; while throughout its whole length, there runs a paved road, unprotected by shade trees, and consequently, its inhabitants were exposed to a more intense heat and drought, than those other places in this quarter. In opposition to this fact, however, we may state, that twenty-five miles north-west of the city, in Butler county, the Epidemic prevailed, for two or three weeks, with frightful mortality, on some of the highest and coolest ridges.

While we thus record the fact, that not only in and around this city, but over the whole cholera region, from south to north, the reign of the Epidemic was cotemporary with the prevalence of summer heat, we are by no means prepared to grant, that this heat was its immediate cause.

The temperature of the past summer, was not remarkably elevated, being only two degrees above our mean summer heat, and if it had been, other summers have been of exalted temperature, but were unattended by Cholera. There must, therefore, be some other cause than atmospheric heat. As to rain, and other forms of humidity, they were not very remarkable. In regard to the summer fruits, it may be said that they were never before so scarce, since the infancy of the settlements of the West; for, on the 26th of April, there was a frost of such severity, as to destroy them nearly all. This uncommon depression of temperature cannot, however, be assigned as the cause of the subsequent Epidemic, for it was of short duration, and only a higher degree of one of the cold nights which annually occur in April and May. Finally, we cannot look to poisonous impregnation of the waters, for the cause, seeing that this could not happen suddenly to the springs, and wells, nor to the river; and, still further, that the people of different villages, drinking alike the waters of the latter, were very differently afflicted by the Epidemic, and some escaped entirely.

It deserves to be noted, that the state of Kentucky, which suffered so fatally last year, has this summer remained exempt from an attack; while Ohio, which last year, was less afflicted than Kentucky, has, this season, been visited extensively, and in some places severely.

It is, also, worthy of remark, that the negroes of Cincinnati, a numerous body, who were greater sufferers than the whites, in 1832, but less afflicted in 1833, were this year comparatively exempt; although, in general, occupying depressed and filthy portions of the city, and living in small crowded houses, on gross miscellaneous food.

The subjects of the disease, in the late invasion of Cincinnati and its vicinity, were chiefly women, of various ages, up to seventy. But many children of both sexes, and a considerable number of men, young and old, likewise perished.

In the town of Fulton, it fell heavier upon the poor than those in comfortable circumstances, many of whom, however, were taken off.

As to the intemperate, some of them died, but not a greater proportion, than of the abstemious, which might be inferred from the fact just stated, that more women than men were destroyed.

In the majority of cases, the disease could not be traced to any exciting cause, or, if one was assigned, it was of a kind to which other persons were constantly exposed with impunity.

### Type of the Disease.

We have said, on a former occasion, that we know of no disease, the symptoms of which are more uniform, or the type better defined. After having seen it three times epidemic, we repeat the same observation. We do not say, that the symptoms of 1834 were a stereotype of those in 1833 and 1832, but the ague and fever of the same spot, generally differs as much, in successive autumns, in its phenomena and the indications of cure founded upon them, as the Cholera has in its successive visitations at this place. The greatest variation of the late, from the preceding attacks, considering them pathologically, was that the type was, on the whole, attended with a more pholgistic diathesis than the others, especially that of 1832.

### Cholerine.

A great number of persons were affected with the diarrhœa, to which the French have given the name of cholerine; and many had an irritable state of the stomach and bowels, which rendered them uneasy under the least irregularity in diet and regimen. This diarrhœa was the epidemic in its mild form. When these attacks were neglected, or the patient was imprudent, they often advanced to full developement, and proved fatal.

### Its Relations to Endemic Cholera.

Cases of common cholera morbus occasionally presented themselves, particularly in May and June. They were distinguishable from Epidemic Cholera by the feculent and bilious evacuations, a greater degree of fever, and less muscular debility. They were generally excited by some irregularity in diet. Instances of cholera infantum were also common, and were distinguishable by nearly the same symptoms. Several adults and children, seized as it were with the *endemic*, finally died with symptoms of the *epidemic* cholera.

### Epidemic Dysentery, and its combination with the Cholera.

The most interesting aspect under which we can view the late epidemic, was its combination with dysentery. The latter preceded the former, and continued to occur after it. For many years that disease has not been as prevalent in this region, as for the last three months. In the majority of cases, the two complaints were uncombined, and each displayed its characteristic symptoms. But in a great number, they were blended in a most striking manner. Thus, of two patients, seized with diarrhœa, one would presently have cholera, and the other dysentery. A patient would be attacked with the latter in the form of tenesmus with bloody discharges, and in two other days, they would become serous, cramps would supervene, and collapse terminate his career. On the other hand, the complaint would set in as cholera, that is, with watery diarrhœa, nausea, thirst, and incipient cramps, and end at length, in dysentery. Again, the patient would have mucous and bloody, in alternation with gruel-like excretions; and in one case, attended by Dr. Shultz, in Fulton, the discharges were copious and thin, but highly colored with blood. Whenever the dysenteric type predominated, or the tendency was to mucous and bloody, instead of serous secretions, the patient was found to be safer, than when the tendency was the other way. As to the diathesis, it was more inflammatory, in proportion as the dysenteric symptoms prevailed, and a great majority of the cases of that malady were so phlogistic as to require the lancet, often two or three times. Did this difference in the condition of the sanguineos system in the two diseases, arise from the depletory and exhausting effects of the copious secretions in cholera? This was probably the fact. In both maladies, the stomach, compared with the bowels, was but little affected. This was particularly the case in the dysentery; but even in the cholera, full and repeated vomiting, was less frequent than in the epidemics of the two preceding years, a modification which probably resulted from the influence of the cause which produced the dysentery, as in that malady, the stomach is generally tranquil. In both of these diseases the function of the liver was affected, nearly in the same manner, and to the same degree—that is, it was suspended. The appearance of bile, by vomiting or stool, except when produced by medicine, was extremely rare, and always indicated a mild attack. Developed fever was more common in the dysentery, than the cholera; as might be inferred from what we have just said on the state of the sanguiferous function; and the manageableness of the case was generally in proportion to the intensity of the fever. In several instances the fever became predominant, and assumed to a considerable degree, the nature of our autumnal remittents; although its paroxysmal character, was not, in general, well marked; it did not recur with a chill, and the copious secretions which accompany autumnal fever, were not present.

### Cause of the Combination of these Maladies.

This blending of the Epidemic with our well known summer endemics, is a curious, though not an unprecedented phenomenon. It would seem, *prima facie*, to indicate, that the whole depended on one cause, modified in its power or its *modus agendi*. If so, a discovery of the cause of our *endemics*, would involve that of the Epidemic. But who can undertake to assign the cause or causes of the former? It is common to say, that they arise from malaria, developed at the earth's surface; but they often prevail on dry limestone ridges in the country, where the *materiel* for foul air—decomposable matter—is almost wanting. Let us assume, however, that they originate from miasmatic exhalations of that kind, and proceed to solve the problems of their connexion with the Epidemic. It is, then, identical with them in its symptoms and nature, or it is not. If identical, why is it recognized every where as a new and terrible malady; and why did it appear in Cincinnati in the year 1832, at least two months after those endemics had ceased? On the other hand, if it have peculiar and distinguishing features, what other cause is it, that modified the cause of our endemics to such a degree as to produce, the pestilence, which all admit to have an existence? Until we can assign *this* cause, we state a fact, and do not give an explanation—we admit a connexion without accounting for it. In fact, we *assume* that our ordinary endemics arise from malaria, and then *assume* that another cause modifies that, so as to make it produce the Epidemic; which at last is equivalent to an admission of total ignorance.

### Treatment of the Cholera.

### *First Stage.*

We begin by reiterating from the two last years, that the Cholera is a curable disease in its early stages only. When it exists as cholerine, (chólericula or slight cholera) it may always be arrested. But unfortunately this has been regarded as a different disease from *the* Cholera; and the people have too often supposed it might be neglected with impunity. The infatuation on this subject, amount every where to a kind of madness, or more properly, idiotism. It is not uncommon to see persons affected with diarrhœa or cholerine, entirely neglecting the means of cure; and, at the same time, extremely anxious to do every thing in their power to avert the Epidemic. When such patients are told, that the fatal malady is already forming in their systems, they are incredulous, and very often cannot be made to believe, that so malignant a disease as that which they dread, can approach with so little display of the armature of death. When they are earnestly and affectionately urged to lie by, and take medicine, they often refuse; and sometimes even think that the physician is governed in his generous advice, by motives of a sordid character! They are beguiled by the malady itself, and allured on to destruction. This singular state of mind, might be supposed to prevail only among the ignorant and heedless, but such is not the fact, for every one must have witnessed it among the intelligent, and we have even seen physicians themselves, thus mystified. The fear of being branded with cowardice, has closed the eyes of many to the danger of suffering the disease to advance beyond this early stage: a dislike to acknowledge themselves sick, and an aversion to medicines, have had their influence on others: finally, a repugnance to withdraw from business, and lie down, while they felt able to go abroad and keep employed, has contributed to the same disastrous result. As we said the two last summers—and to repeat in every form of expression, and on all occasions the designation of cholerine or diarrhœa, as the *premonitory* stage of cholera, instead of the *first* stage, has contributed, beyond all human calculation, to the mortality of the Epidemic. That single word *premonitory* has, indeed, slain its tens of thousands, in every country where the cholera has appeared? and will, it is to be feared, continue its ravages, till the pestilential constitution dies away. We would loudly and earnestly call on all who may read these pages, to unite in banishing it from the nomenclature of cholera history; and beseech every member of the profession, to insist on the great and momentous truth, that the symptoms to which it has been applied, are those of the pestilence itself, and that all who labor under it, have already felt the impress of the secret poison, and are liable at any moment to become seriously ill and die.

In this stage, as we have repeatedly said, the Epidemic *may* be cured in ninety cases out of a hundred—but let it advance beyond this stage, to vomiting and cramps, and ninety out of a hundred may die. A single day's delay, a night of watching with a sick friend, a hearty or indigestible meal, an hour of grief, or a wetting of the skin, may be sufficient to develope the fatal stage. The physician who directs his attention upon these cases, will save the lives of multitudes, but may get no credit for curing *Cholera*. The recovery, under his hands, of one patient affected with vomiting and cramps, will excite the admiration of the populace, more than the timely preservation of a hundred patients afflicted with the early symptoms only. Such are the ignorance and perversity of the people; but it is his duty to disregard both, and insist on the indispensable necessity of meeting the disease while it may yet be conquered.

The treatment of this stage, in the late visitation, was nearly the same as in the preceding. When the patient had an active pulse, (not being exhausted by the long continuance or the profuseness of the diarrhœa,) bloodletting was required, calomel was always necessary, and by all odds, the most efficient of our remedies. Opium, stimulants, antacids, and sudorifics, were also beneficial. But nothing could be relied upon, unless the patient quit eating solid food, and laid down. By these simple means the victory was achieved—but it brought no crown of laurel, nor very often called forth an expression of gratitude.

### *Second Stage.*

We shall call that the second stage, in which vomiting and cramps or spasms, were present. In this stage, the alvine discharges were always serous. It often followed on a diarrhœa, or first stage, of several days duration; at other times, the two symptoms just mentioned, were present almost from the beginning; and Dr. Wood has mentioned a patient to us, who became affected with cramps in his legs, while his stomach and bowels felt perfectly sound; he died, however, in a few hours.

The treatment of this stage did not differ materially from that of the first, except in degree. Great energy was requisite to secure a favorable event. Bloodletting was often, not always, necessary. After the lapse of even a few hours, it was contra-indicated by the exhaustion of the patient. The cases which bore the largest bleedings, as already intimated, most frequently terminated well. Local bleeding was not used, as far as I heard; and, indeed, the state of the skin would seem to render cupping almost impracticable. The *great* remedy in this stage was calomel—we mean that it was the *great reliance.* Every physician made it the basis of his treatment. It was given in doses of from 20 up to 60 grains—rarely in greater; and repeated, till, in some instances, several drachms were administered. It was often combined with opium, but less of the latter was required than in 1832, for the type of the disease was more inflammatory. We sometimes combined it with large doses of powdered aloes, but it was more commonly united with camphor or capsicum, or both. It had been repeatedly given in such connexion, the two preceding years; but the alleged success of Dr. Cartwright, of Natchez, from the use of the triple compound, evidently led to a more liberal use of the two latter articles, than in the previous attacks. In our own practice, but more especially in the practice of Dr. Towler, and Dr. Shultz, in Fulton, we saw this compound repeatedly fail, and finally, were not convinced that it was, in most cases, more efficacious than calomel alone. The experience of so able a practitioner as Dr. Cartwright, is entitled to great consideration, and we are not disposed to entertain or express a doubt of what he has reported on that subject; but his practice seems to have been chiefly among the negroes of Mississippi; and their constitutions, it is well known, require more stimulation than ours; we can readily conceive, then, that he found much efficacy in the union of capsicum and camphor with calomel. We have been favored with a paper by Dr. Yeargain, of Princeton, in the same state, in which he gives the results of his experience, with this compound in the following language:

"The treatment the writer believes most effectual, and which he has settled upon, after a trial of all the remedies he ever heard recommended, is, to bleed, if the patient is seen in time to expect reaction can follow the remedy; if not, to give immediately sixty grains of calomel, ten grains of powdered camphor, and ten grains powdered pepper, to be washed down with half an ounce of *paragoric elixir* undiluted, the table spoonful of this camphorated tincture, may be repeated in a short time, if the patient does not grow warm, and the vomiting and purging cease; the calomel powder may be repeated in two hours; and the patient made to drink of ginger or peppermint tea, no other drink allowed; these remedies to be assisted by warm applications, warm bath, brisk frictions, with a warm mustard poultice over the whole of the abdomen. There must be no solicitation about the operation of the medicine. The bowels will wait until even the next day, if the morbid action is overcome."*

The first effect of the calomel alone or combined, when it did good, was to quiet the stomach, and arrest the secretion and excretion of serous fluid from the bowels. The second was to excite colored discharges, which were green or yellow—rarely the last, until after the former had ceased. When these colored discharges appeared, the patient was said to be safe; but we saw three cases in which he passed into fatal collapse, with dark bilious excretions, and died. When the serous discharges began to abate, the restoration of the biliary secretion was often accelerated by the use of senna, scammony, the blue pill, rhubarb, aloes, and other drastics, which rendered the further exhibition of calomel unnecessary. When the patient was greatly exhausted, or of a phlegmatic temperament, these intestinal irritants were more necessary to the restoration of the secretions of bile, than when he was in a vigorous and phlogistic condition.

With the occurrence of colored secretions, the spasms, if not previously subdued, generally ceased. For the purpose

* We at first intended to publish this gentleman's paper, but concluded, at length, to limit ourselves to this extract. The presentation to Dr. Cartwright, of a beautiful silver vase, by certain planters in the neighborhood of Natchez, is a strong evidence of his success, and an act equally honorable to all the parties concerned.

of removing them, much less of direct effort was made, than in the two former visitations. The warm bath, and hot applications, sinapisms, blisters, and frictions, were not so often employed, and, we are disposed to think, with no injury to the sick. A perception of their comparative inefficacy, probably led to some abatement in their use.

Thus, experience in this, as in other cases, simplified the treatment, and reduced the number of therapeutic agents.

*Third Stage.*

The symptoms of collapse, in the cholera of the present year, were nearly the same with those of the preceding. Of the treatment of this stage, we have but little to say. We have repeatedly inquired of our professional brethren, by what means they succeeded in curing it; and were invariably answered, that they knew of no remedy for it; which was in exact coincidence with our own observation. Indeed, all who fall into collapse, should be expected to die. If any have recovered from that condition in Cincinnati, they have not met our notice. We do not deny but that recoveries may have taken place; but from the total inefficacy of all the means we have seen employed, we feel justified in saying, that the number of such results has been small indeed. We saw two cases in which the patients distinctly emerged from that state; but they both died of consecutive fever, with signs of diseased brain; and, also witnessed several instances of partial resuscitation, but the patients fell back into collapse, and were lost. We cannot, therefore, but look with indifference on the immense farrago of means for the cure of this stage; and with dismay at the volumes that have been written on its pathology and indications. They seem to us to have been worse than useless; for, by holding out a delusive prospect of cure in the last, they have diverted the minds, both of the people and the profession, from the first or curable stage.

### Treatment of the Dysentery.

The treatment of this disease was substantially the same as that of the Cholera. It required the lancet much oftener, and the blood drawn was more frequently covered with an inflammatory crust. The effect of bleeding was almost always good; and in no instance that fell under my observation, prejudicial. As in cholera, after bloodletting or without it, when not required, the chief reliance was on calomel; it was not given, however, with stimulants and aromatics, but alone, or in alternation with castor oil, or that medicine in connexion with mucilage of gum arabic and sugar. From the tormina attendant on many cases, opium was imperiously demanded. In some instances, Dover's powder, or opium and ipecac were found better than the opium alone. Inflammatory action being moderated by the lancet, nothing was more efficacious than the combination of opium and calomel.

The former soothed the pain and spasm of the muscular tunic of the bowels, the latter excited the liver to renewed secretion, and superseded the irritation of the mucous membrane of the colon and rectum. As in cholera, whenever bilious or atrabilious discharges occurred, the patient was found to be improving; but the muco-sanguineous secretions sometimes recurred, and in a few cases, continued for a while to alternate with the bilious, so that immediately after a discharge of mucous and blood, there would occur one of bile. In one case, that of a lady, who had long been affected with liver complaint, the discharges put on a purulent aspect, and were for two or three days, so copious, as to render it probable that an abscess of the liver might have been emptied into the bowels. In the course of a few hours, this patient would have several discharges of blood and mucous, unmixed with bile—then of bile, blood, and a secretion of a purulent aspect. The pain, moreover, was not confined to the rectum, but extended into the right hypochondriac region. Bloodletting, calomel, ipecac, opium, mucilages, active cathartics, blisters, rhubarb, prepared chalk, enemata, the bark and the tinctures of kino and catechu, were among the remedies, which in the progress of this tedious and painful case, were employed; several of them appeared to do good, but the discharges were still frequent, vitiated, and attended with great pain, and much exhaustion of strength, when we resorted to the acetate of lead in combination with opium. The effect was most happy. The medicine was given in three grain doses, every three hours, and before the first 12 grains were administered, her amendment was decisive. When 36 grains were given, the midicine was discontinued, and she was put on the use of tonics and diet, with chalk and opium occasionally. In several instances, we saw a decoction of bark of unquestionable advantage. Dr. Ridgely informs us, that he obtained very beneficial results in a number of instances, from liberal doses of rhubarb and magnesia. Dr. Richards arrested a number of cases, in the *beginning*, with one or two doses of the spiced tincture of rhubarb and paregoric. Dr. Dodge, in one most obstinate case, abated the tenesmus and copious bloody secretions, and saved his patient, with a decoction of crow-foot root, (*geranium maculatum.*) Injections of starch and laudanum often did good; but a hip bath was on the whole still better.

As we shall follow this article with another by Dr. Bailey, on the Dysentery, as it prevailed in the neighborhood of the city, we shall not extend this section, but proceed to state, in conclusion, a number of

### Miscellaneous Facts and Cases.

---

*Cure by Sweating.*

1. I was assured by a poor intemperate man, in Fulton, that being attacked with Cholera, he was advised to go to bed and drink freely of "elder berry tea," (*sambucus nigra,*) with whiskey; which he did; and a profuse sweat coming on, he immediately got better. Sometime afterwards, losing his wife with the same malady, he continued on his feet all the night and through the forenoon, with some degree of diarrhœa upon him; passing into a state of collapse in the course of the following night, he died the next day. We do not entertain much doubt that if he had been treated as early and in the same manner, in the second attack as in the first, he might have been saved; and state the case, not merely to animate others to a timely resort to medicines; but to show the efficacy of a decided sudorific treatment in the beginning of the disease.

*Convulsions.*

2. A little child, in the practice of Dr. Towler, in Fulton, during the spasmodic stage of the disease became hemiplegic, and died with convulsions. This often happens in cholera infantum.

*Serous Uterine Secretion.*

3. When the disease was at its height in that town, a woman was delivered, having a natural labour, and appearing quite comfortable after the event. In a few days it was observed that the lochial discharge was increasing, instead of diminishing, and that the patient was failing in her strength. In its character the excretion was decidedly serous, and at length became exceedingly profuse. When we were called into consultation with her physician, Dr. Towler, her pulse and the heat of her system were reduced, and her lips were purple. In short, she passed into highly characterized collapse, and died. The uterine secretion was manifestly choleric, and vicarious of that from the bowels. As far as we recollect, the case is a new one in kind; and ought to serve as a warning to those who have obstetrical practice during the prevalence of the Epidemic.

*Bad Effects of Mineral Water.*

4. A patient of Dr. Shultz, in the same town, which I likewise saw in consultation, had been more or less affected with diarrhœa, when he visited the epsom salt spring, near Newport, on the opposite side of the Ohio, and drank freely of the water. It operated copiously, and he was soon after taken with cramps, and passing into collapse with frightful rapidity, died in a few hours after the Doctor was consulted.

*Imperfect Dislocation of the Lower Jaw.*

5. In the same town, a casualty occurred to a choleric patient, which excited considerable attention. An elderly woman, in vomiting, suffered what Mr. Cooper has described as an "imperfect luxation" of the under jaw. Her physician, as the jaws were but little separated, regarded it as tetanic. We were not called into consultation, but by permission, saw the patient, *en passant*, with her jaws bound up, and not making an examination, presumed, that it might, in fact, be a case of rigidity, connected with the spasmodic stage of the cholera. Subsequently, no change having taken place, the idea of its being a dislocation, was started in the neighborhood, and the attending, called in a consulting physician, who reduced it.

The latter, with his medical friends, naturally commented on the fruits of this hasty, (or rather unperformed) observation, with the same severity as if the patient had been our own, and subjected to a full and special inspection. Of this we are far from complaining; for the cause of humanity requires, that carelessness, even in concurring with others, should be punished by injury of reputation; and the older the physician, the less should he be excused. Indeed society needs every kind of protection against the effects of inattention, inaccuracy and ignorance; and we can scarcely regret the industrious promulgation of this occurrence, when it affords us so fine an opportunity of impressing upon our *young* readers—they for whom we write, and who are the hope of the rising generation—the great importance of patient and persevering inquiry, into every case with which they may have the least connexion. A habit of sound and perspicuous observation, is as necessary to the support of a good medical reputation, as to successful practice; for the mistakes of a physician become the common property of the profession, and are turned against him by the guardians of its honor and interests, as a means of exciting others to greater care and deeper study.

*Extraction of the Cataract followed by Collapse.*

6. About the time the Epidemic began to manifest itself in this city, a poor and aged woman, from a neighboring state, presented herself at the *Cincinnati Eye Infirmary*, with cataract. The extraction of the lens of one eye, (without accident, and the cornea healing kindly,) was followed by debility and nervous irritation. After the lapse of some time, during which she was confined to the bed, she sunk imperceptibly into a kind of collapse, and expired. We are of opinion, that the choleric poison exerted a fatal influence on the system of this elderly woman, previously feeble, and still further enervated by the operation; and mention the case, to show the impropriety of performing any surgical operation during the prevalence of Cholera, that is of a nature to be postponed.

*Obscure and Irregular, but Fatal, Cholera.*

7. A gentleman, from Louisiana, came to Covington, opposite Cincinnati, after the Epidemic had ceased. He was somewhat indisposed when he arrived, and employed Dr. Reynolds. We saw him, in consultation, several times. His malady was not well defined; but on the whole presented the symptoms of that kind of functional derangement in the abdominal viscera, and that state of the feelings and faculties of the mind, which are present in, and constitute hypochondria. He took emetics, calomel, the blue pill, aloes, colocynth, and other medicines of a chologogue and drastic kind, which operated rather sparingly. After an illness of more than a week, his pulse gradually became imperceptible, his skin purple in spots; his fingers sodden; his nails blue; his voice sunk to a whisper; but he retained his senses and a considerable degree of muscular strength. He had no cramps, but the predominant symptoms of collapse were present, and in 12 or 14

hours after his pulse ceased, he expired. If not the sole cause of this malady, the choleric poison evidently exerted a fatal influence in this case; which was not the only one of a like kind that fell under our observation; and may be mentioned among the anomalies, which the physician should not allow to impose upon him, by their resemblance to other and milder diseases.

*Cholera supervening on Intermittent Fever.*

8. A physician, returning from New Orleans, was seized with intermittent fever, and stopped in Fulton, two miles above Cincinnati. When convalescent, the Cholera became violent in his neighborhood. In the latter part of the night, he was seized with profuse watery discharges; his pulse failed, his eyes sunk deep in their sockets, his face became pale, and his countenance choleric. Being a physician, he took, before morning, a large quantity of calomel, laudanum, and brandy, which arrested the discharges; and before night, bile appearing as they were reproduced by the calomel, he felt much better, and under the conjoined use of tonics and mercurials, got well.

*Another Case.*

9. A patient of Dr. Walker's, whom we saw in consultation, had labored under intermittent fever, from which he was not restored, when he was seized with Cholera. Large doses of calomel and aloes brought away free discharges of dark colored bile, but while they still recurred, he sunk into collapse, and was lost.

*Intermittent Fever, Dysentery, and Cholera.*

10. We incidentally saw a poor woman, on the hills near Cincinnati, and prescribed for her several times, who labored, in succession, under the forms of disease just named. After having for a number of days been affected with a quotidian intermittent, a violent dysentery supervened. It was in this stage that we saw her, and found her alvine excretions bloody and mucous, with tenesmus. Liberal doses of calomel and Dover's powder corrected this state of the secretions, and for a short time she seemed likely to recover, when the characteristic diarrhœa of Epidemic Cholera set in, and, as we understood, she died with the usual symptoms attendant on the closing stage of that malady.

*A Case of Cholera, running through all its stages, uninterrupted by Medicine.*

11. A middle aged woman, in Fulton, was seized with Cholera, and her husband sent for Dr. Towler, who, however, could not prevail on her to take medicine. When we saw her first, by permission of the Doctor, she was passing into collapse, and at a second visit, was nearly pulseless, with purple lips, shrivelled fingers, cold extremities, and feeble and husky voice. Being a fanatic of the Mormon sect, she said *her* Bible ordered those who are sick to resort to herbs. About the time when she was sinking into collapse, a sister fanatic sent her word, that she had received a special message from Heaven, saying—Let the sick sister drink a tea of dog-fennel and arsmart, (*anthemis cotula et polygonum persicaria.*) This was done, and as these are both acrid plants, the infusion probably sustained her through the collapse, out of which she rose, when we looked for her to die. A dose of calomel, however, administered by stealth, may have contributed to the result. Secondary, fever now ensued, and after a tedious course of eight or ten days, she died with strong signs of disease in the brain. During this fever, she consented to let Dr. Towler prescribe for her. In this case the encephalic disease was not the consequence of the previous administration of either stimulants or narcotics.

*Slaughter houses not pernicious.*

12. Most of the butchers' shambles are near each other in the valley of Deer creek, which has an atmosphere thoroughly impregnated with the various exhalations that arise from putrid blood and other offals; but as far as we could learn, nearly all the operatives and inhabitants of the valley escaped both the Cholera and the Dysentery.

*Mortality in Fulton.*

13. Much was said about the great mortality in Fulton, amounting, as we have already stated, to near four per cent.; and it was even said, that the physicians who practised there, were unskilful. We had, however, much opportunity of knowing what practice they pursued, and can state, that it was substantially the same as that pursued in the city and elsewhere. The causes of great mortality in that open village, were the same that are operative elsewhere. In the first place, many of the inhabitants were recent emigrants, who had arrived since the disease prevailed the year before, from places where it had not been epidemic, and consequently they were new and liable subjects. Secondly, a great proportion of those who died were poor persons, badly provided with comforts and early medical aid. In one family, that fell under our own observation, four out of seven died, in a destitution of medical aid, that was nearly absolute. Thirdly, not a few entirely neglected to avail themselves of the experience of the *profession*, and sought relief from the recipes of *empiricism*. Fourthly, as is usual, a great proportion, even of those who placed reliance on the profession, deferred, an application for relief, till the curable stage of the disease had passed by. These were the true causes of the mortality, and quite sufficient to account for it.

*Mortality in Cincinnati.*

14. The aggregate mortality in the city was small, because but few were attacked. The proportion of deaths to the number of cases, was, *perhaps*, nearly as great as in the preceding visitations. By the bills of mortality, the whole number of deaths did not exceed 150; being little more than a half of one per cent. of the whole population. The *three* epidemics have destroyed about four per cent. The week of the greatest mortality, was in the week ending July 30th, during which, the number of deaths were about four a day.

*Mortality in our own Practice.*

15. In our own practice, in the city, we lost two patients—A man who manifested no signs of Cholera till 24 hours before his death, although indisposed with gastric and intestinal *malaise*, as we understood from him for 5 or 6 days previously; and a child, seven months old, whose disease commenced as ordinary cholera infantum, and ended with the serous discharges, unquenchable thirst, restlessness, and exhaustion, which characterize the final stage of Epidemic Cholera. In the country, we lost two patients—a child and a woman. The child died in collapse, with *bilious* discharges; the woman we saw once only. The disease appeared to be awakened in her by the anxiety of mind, induced by the departure of her husband for New Orleans; a fact which we mention, to show the variety of exciting influences, which may give activity to the remote cause.

*Relative prevalence of Cholera and Dysentery in different places.*

16. These two diseases were not greatly prevalent in the same place, and where one was general, the other was not. The Dysentery has reigned much more extensively in the West than the Cholera, but has not attracted so much attention as the latter.

*Comparative Mortality of Cholera and Dysentery.*

17. The Cholera was decidedly a more fatal malady than the Dysentery; and most that died of the latter, had choleric symptoms and collapse, in the final stage. In the city, we lost no patient with dysentery: in the country one—a lady, who appeared to have labored for some time under scrofula of the mesenteric glands, with repeated attacks of mesenteritis.

*Effects of sudden Changes of Weather.*

18. When the disease had nearly ceased, a sudden change of weather re-produced several cases, which, however, were not followed up by others. The same thing was observed last year. Indeed, the occurrence of rain, while the complaint was still prevalent, seemed to be attended with prejudicial effects, as it was preceded by great heat, and followed by a reduction of temperature—first augmenting, and then suddenly checking perspiration.

*Present State of Health in the City and Country.*

19. At this time, Sept. 18th, the city is healthy, (although now and then a death occurs from Cholera,) and the immediately surrounding country not less so. In the former, there is very little autumnal fever: in the latter, intermittent fever exists to a considerable extent, in some of the alluvial valleys, where neither Cholera nor Dysentery had prevailed this year.

*Diseases of Animals.*

20. There has been no epizootic disease, in the city or neighboring country, that does not occur every year. As stated in our last number, the locusts (*Cicada septendecim*) were numerous in the month of June; but with this exception, the insect kingdom has exhibited no phenomenon of any kind; nor has the state and progress of vegetation been in any circumstance remarkable.

*Diminished Cholera-phobia.*

21. The alarm in Cincinnati and the surrounding country, upon the development of the disease, was far less than in the year 1832, or even 1833, and to this may perhaps be ascribed, in part, its lesser mortality. Of contagion there was, among the people who witnessed it before, not the smallest dread. They often, indeed, crowded round the cold, blue, and expiring patient, in such numbers as to require the interposition of the physician.

*Cincinnati, Sept.* 18, 1834.

